# Cytokines and MicroRNAs as Candidate Biomarkers for Systemic Lupus Erythematosus

**DOI:** 10.3390/ijms161024194

**Published:** 2015-10-13

**Authors:** Barbara Stypińska, Agnieszka Paradowska-Gorycka

**Affiliations:** Department of Biochemistry and Molecular Biology, National Institute of Geriatrics, Rheumatology and Rehabilitation, Warsaw 02-637, Poland; E-Mail: kancelaria@ir.ids

**Keywords:** SLE, biomarkers, microRNA, cytokines

## Abstract

Systemic lupus erythematosus (SLE) is a systemic autoimmune disease, with varied course and symptoms. Its etiology is very complex and not clearly understood. There is growing evidence of the important role of cytokines in SLE pathogenesis, as well as their utility as biomarkers and targets in new therapies. Other potential new SLE biomarkers are microRNAs. Recently, over one hundred different microRNAs have been demonstrated to have a significant impact on the immune system. Various alterations in these microRNAs, associated with disease pathogenesis, have been described. They influence the signaling pathways and functions of immune response cells. Here, we aim to review the emerging new data on SLE etiology and pathogenesis.

## 1. Introduction

Systemic lupus erythematosus (SLE) is a heterogeneous autoimmune disease of multifactorial etiology with extensive multi-systemic effects. In SLE, the immune system is misdirected against a wide range of autoantigens. Immune effectors trigger signaling pathways causing some disease-specific tissues damage. The most characteristic symptoms that allow recognition of the disease include: skin lesions (malar rash, discoid rash), sores in mouth or nose, musculoskeletal manifestations as arthritis, arthralgia or myositis, bone fragility fractures and secondary pain amplification [1]. Currently the diagnosis is based on American College of Rheumatology (ACR) criteria. Simultaneous presence of four (or more) out of eleven criteria allows the identification of the disease.

SLE is classified among systemic autoimmune disorders because of the presence of autoantibodies such as: antibodies directed against double-stranded DNA (anti-dsDNA), anti-small nuclear RNA-binding proteins, anti-phospholipid antibodies (aPL) or anti-Smith (anti-Sm) nuclear antigens in abnormal titer. Anti-dsDNA Immunoglobulin G (IgG) is an important disease marker, given that it is detected in the great majority of patients’ sera, prior and after diagnosis. Anti-ds-DNA antibodies measurement, besides being used in the diagnosis, helps to monitor the progress of patients and to study the pathogenesis of the disease [2]. Other biomarkers, currently used in diagnosis and monitoring of SLE, include acute phase proteins, erythrocyte sedimentation rate (ESR), C-reactive protein (CRP) and complement proteins level [3]. Unfortunately, so far there is no serologic test that reliably and accurately measures disease activity [4].

The complex etiopathogenesis and heterogeneous clinical manifestations make SLE commonly misdiagnosed. This diversity is a reason why a treatment may have beneficial effects in only a subset of patients. Identifying new SLE biomarkers might be a useful tool to sub-classify patients, quantify the risk of organ involvement and predict which clinical manifestation they might develop, as well as assign them the most appropriate treatment. It may also facilitate early and accurate SLE diagnosis and improve the evaluation of medications in clinical trials. Therefore, finding new specific biomarkers is very important and there is constant effort to obtain new and more valid tests for a better management of the disease.

Cytokines and microRNAs (miRNAs) have shown an early promise as biomarkers for lupus susceptibility, diagnosis and monitoring. Cytokines play a key role in the regulation of the type and magnitude of immune response. There is growing evidence of the pathogenic role of cytokines in the processes that cause inflammation, skin lesions and organ symptoms, associated with SLE. It seems that not a single cytokine, but the altered pattern of these markers altogether, may contribute to the autoimmune processes. Many of them have been investigated as potential SLE biomarkers due to their easy accessibility as well as many convenient and specific methods of their measurement and most importantly due to their pivotal ability to mediate local inflammatory processes and tissue injury [5].

Recently, the list of molecules important for SLE pathogenesis has been extended by miRNAs. These factors comprise a fundamental layer of post-transcriptional gene expression regulation. Patients with SLE have revealed unique miRNA signatures when compared with healthy individuals or those with other diseases. Dysregulation of miRNAs has also been found to be associated with disease activity and major organ involvement [6]. MiRNAs have the potential to act as biomarkers for the diagnosis and assessment of patients with SLE.

The main aim of this review is to recollect and summarize recent evidence supporting the role of cytokines and miRNAs in the immunopathology of SLE, and also to analyze their possible role as biomarkers that would be useful for disease management or evaluation of the activity of SLE and the risk of different organs involvement.

## 2. Cytokines: Functions and Contributions to the Pathogenesis of SLE

The array of studies shows that expression disturbances of certain cytokines are significant for SLE pathogenesis. Imbalance between pro- and anti-inflammatory cytokines is critical for development of clinical manifestations and organ damage in SLE patients. Among the most important cytokines in disease development are: interferon type I (IFN-I); interleukin-6 (IL-6); interleukin-17 (IL-17); B lymphocyte stimulator (BLyS); A proliferation-inducing ligand (APRIL); and tumor necrosis factor-α (TNF-α) [5]. There are several mechanisms of how cytokines contribute to development of lupus. Particularly worthy of mentioning are those leading to immune cell function-alterations, impaired cell death signaling or osteoclastogenesis and osteoblastogenesis disturbances.

Cytokines imbalance is partially responsible for low activation thresholds of immune cells and their maturation and differentiation disturbances. One of the mechanisms of how cytokines lead to the immune tolerance loss is by contributing to the impaired B and T lymphocytes function. B-lymphocyte activity plays a central role in course and development of SLE. Among cytokines regulating B cells activities are BLyS, APRIL, IFN-I and IL-6.

BLyS is one of the most important cytokines governing B cells functioning. It has a direct ability to induce B cells survival, proliferation, differentiation and immunoglobulin secretion [7]. Its deficiency blocks mature B cells development in mice [8]. On the other hand, BLyS overexpression increases the number of effector T and mature B cells. These cells exhibit prolonged survival and hyperactivity, which leads to the development of autoimmune-like manifestations in mice [9].

Similarly, APRIL is responsible for induction and maintenance of B cell responses. It serves as a potent coactivator to augment Ig production and to upregulate surface expression of B cell effector molecules [10]. These findings highlight the importance of BLyS and APRIL in pathogenesis of autoimmune conditions like SLE.

IFN-I is another important cytokine that governs B cell proliferation, activation, and survival. Not only it increases the level of BLyS, but also directly promotes IgG subtype production. It induces the expression of major histocompatibility complex (MHC) class I and class II antigens and of co-activation molecules such as CD40, CD80, CD86 and more [11]. INF, by up-regulating B cells activities, can cause inflammation and tissue injury. Its exogenous administration accelerates autoimmune disease progression in New Zealand Black (NZB) mice [12,13].

IL-6, which also plays a critical role in the B cells hyperactivity and immunopathology of SLE, may have a direct role in mediating tissue damage. IL-6 takes part in B cell to plasma cells maturation and promotes IL-21 secretion by CD4 T cells, which contributes to antibody production by B cells. IL-6 receptors (IL-6Rs) are constantly expressed on B cells from SLE patients in contrast to B cells from healthy controls [14]. Moreover IL-6 is capable to indirectly facilitate the activation and expansion of B cells by down-regulation of CD5 expression, which suppresses BCR signaling and thereby controls auto-reactivity of B cells [15].

Although B cells are recognized as crucial elements of SLE pathogenesis, it is well known that abnormal T cell function is also significant for that process. Cytokines are crucial for maintaining an appropriate balance between pro-inflammatory and regulatory cells, for which loss leads to the development of autoimmune diseases [16]. Regulatory T cells (Treg) maintain immunological homeostasis, and prevent autoimmunity. In SLE patients, the number of these cells is decreased and their function is significantly disrupted, favoring development of proinflammatory cells and leading to chronic inflammation [16]. For instance, the number of Th17 cells increases. These cells have been associated with the induction of autoimmune inflammation and contribute to the differentiation and proliferation of osteoclast causing bone resorption [16]. These actions are initiated under the influence of IL-17, IL-6, TNF-α, interleukin-23 (IL-23), interleukin-22 (IL-22), granulocyte-macrophage colony-stimulating factor (GMCSF) and some other additional novel factors. The IL-23-IL-17 axis has a central role in the autoimmune inflammation. IL-23 is required for expansion of pathogenic Th-17 cells and is necessary for their maintenance [17]. IL-17 is responsible for inflammatory cells recruitment and facilitates T cell infiltration in the site of inflammation [18]. Interactions between IL-23 and IL-17 are essential not only for the onset phase, but also for the destruction phase of SLE, which is characterized by the T cell-mediated activation of osteoclastogenesis [19]. Excessive presence of these cytokines leads to imbalance between proinflammatory and Treg cells in favor of the Th17 cells in SLE [20].

IL-2 and IFN are also relevant for a proper function and proliferation of T cells. IL-2 is significantly downregulated in SLE patients, and its low level is associated with disruption in activation induced cell death (AICD), reduction in Treg population and decrease in cytotoxic activity of CD8^+^ T cells [21]. On the other hand, IFNα/β stimulates T cells activation by up-regulation of various chemokines production (including CXCL8 (IL-8), CXCL9 (MIG), CXCL10 (IP10), and CXCL11 (I-TAC), and of their receptors) thereby influencing dendritic cells (DCs). In consequence, DCs are stimulated to produce cytokines like IL-12, IL-15, IL-18, and IL-23 [22]. Moreover, IFN enhances T-cell proliferation via a direct mechanism or via IL-15 induction by antigen-presenting cells (APC) [23].

Another mechanism leading to SLE progression is impaired cell death signaling. Apoptosis has been considered as the major source of autoantigens in SLE. Lupus serum has a strong apoptosis-inducing capacity, contributing to increased death of monocytes and lymphocytes [24]. Many studies demonstrate increased level of the apoptotic ligands in SLE: TNF-related apoptosis-inducing ligand (TRAIL), TNF-related weak inducer of apoptosis (TWEAK), and Fas ligand (FasL) [25]. Those findings show that apoptosis is significantly bolstered in SLE patients leading to the accumulation of apoptotic cell remnants and—As a consequence—Increased load of nuclear autoantigens.

IFN is one of the factors contributing to abnormalities in the process of apoptosis. IFN inducible genes induce cell death, decrease viral protein synthesis and promote lymphocyte, neutrophil, macrophage and DC activation. Activated DCs secrete excessive amounts of IFN contributing to its pathological function. Augmented amounts of IFN-α up-regulate levels of Ro52, which was recently identified as an E3 ligase with anti-proliferative and pro-apoptotic properties. In this way, IFN indirectly contributes to the induction of apoptosis in SLE [26].

It has been postulated that in SLE patients, dysfunction of apoptosis could result in an inappropriate longevity of autoreactive B cells and consequently in increased production of autoreactive antibodies. This is due to inappropriate expression of both Bcl-2 and Fas in those cells. Bcl-2 enhances cell survival by inhibiting or delaying apoptosis. Bcl-2 expression is significantly elevated in plasmatic cells of SLE patients. Conversely, Fas is a proapoptotic molecule and it is down regulated in SLE-like autoimmune disease in mice [27]. Interestingly, there are some studies showing that INFα up-regulates expression level of Bcl-2 both *in vitro* and *in vivo*, which further emphasizes the importance of this cytokine in the pathogenesis of SLE [28].

Many individuals, suffering from lupus, have a striking increase in premature atherosclerosis. Abnormal function or decreased levels of endothelial progenitor cells (EPCs) and myelomonocytic circulating angiogenic cells (CACs) are established atherosclerosis risk factors. Denny *et al.* [29] showed that these anomalies may be associated with increased level of INFα in SLE patients. INFα triggers apoptosis of EPCs and CACs and skews their phenotype to non-angiogenic and therefore plays a crucial role in SLE pathogenesis; its overexpression is related to premature artherosclerosis in the disease [29].

Cytokines also lead to osteoclastogenesis and osteoblastogenesis disturbances. The ability of bone marrow mesenchymal stromal cells (BM-MSCs) to differentiate into osteoblasts is limited in SLE. Tang *et al.* [30] reported that those functional disturbances are probably related with TNF, which, by activation of NF-κβ signaling pathway in SLE-BM-MSCs, inhibits the BMP-2-induced osteoblastic differentiation and may cause osteoporosis in SLE patients [30]. TNF also induces osteoclastogenesis either by promoting proliferation of osteoclast precursor cells or by participating in activation of the differentiated osteoclasts through the RANK/RANKL signaling pathway [31,32].

Many studies showed that exogenous IFNα immunostimulation leads to various autoimmune manifestations. Almost every case of patients treated with IFNα is associated with increased production of autoantibodies, most notably antinuclear antibodies and anti-dsDNA antibodies. Clinical lupus develops in about 1% of these patients. Cessation of IFNα therapy results in reduction in the titer of these antibodies and subsequently in a significant clinical improvement or even remission of SLE. The above only further confirms the importance of this cytokine in the pathogenesis of lupus [33].

Summing up, cytokines play a very important role in SLE pathogenesis. The level of their expression has a strong influence on the immune system proper activity. Any abnormalities may cause disturbances at various stages and in many different ways. All those facts strongly indicate that cytokines are interesting, potential candidates for lupus biomarkers.

## 3. MicroRNA-Functions and Contribution to the Pathogenesis of SLE

MiRNAs represent a large family of endogenous noncoding RNAs that comprise a fundamental layer of post-transcriptional regulation of gene expression. Those small, evolutionary conserved noncoding RNAs bind to complementary sequences of their target mRNAs within the 3ʹ untranslated region (3ʹ UTR). MiRNAs negatively regulate protein expression at the post-transcriptional level through reduction of mRNA stability and inhibition of translation [34]. MiRNAs as well as cytokines, participate in the regulation of innate and adaptive immune responses. The dysregulation of miRNAs in SLE could be result of multiple environmental factors. Since miRNAs can regulate every aspect of cellular activity from differentiation and proliferation to apoptosis, alterations of their expression contribute broadly to various aspects of lupus pathogenesis. Therefore, the epigenetic mechanisms are a window to the understanding of the possible mechanisms involved in the pathogenesis of complex diseases such as SLE [35]. While characterization of miRNAs expression patterns in patients with SLE can have potential diagnostic use, new discoveries in cell type-specific miRNA expression profile, during the disease progression, may provide further understanding of SLE immunopathogenesis.

**Figure 1:**
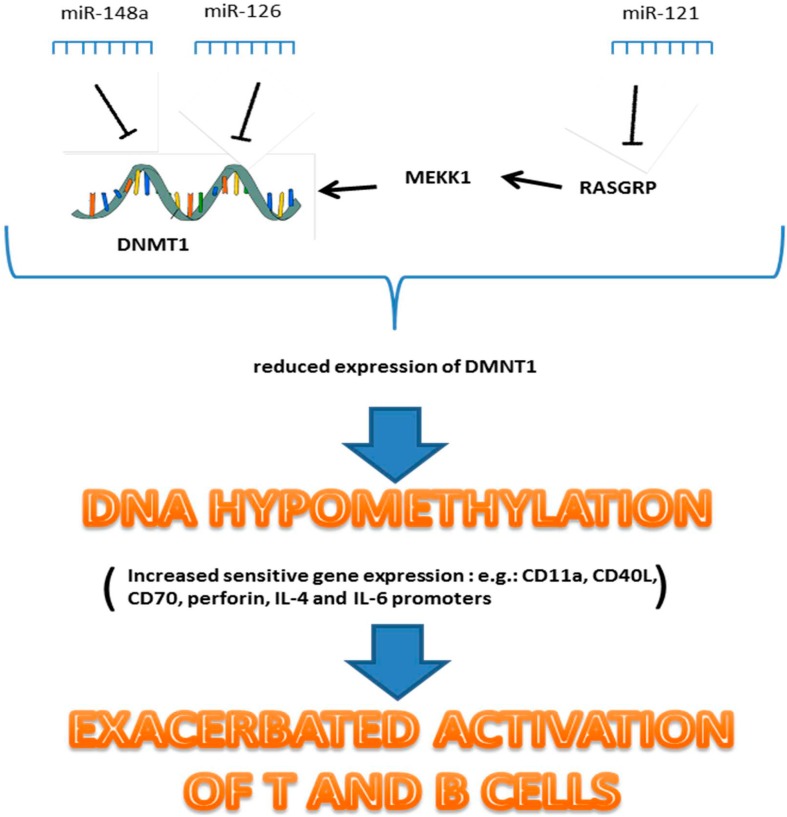
MiRNA involved in epigenetic deregulation in SLE.

T cells of SLE patients exhibit a global reduction in DNA methylation. DNA hypomethylation correlates with disease activity in lupus patients. This epigenetic deregulation can lead to an exacerbated activation of T and B cells [36]. Recent works suggest that miRNAs are involved in regulation of DNA methylation in lupus. Three different miRNAs, targeting DNA methylation machinery in SLE, were successfully identified (Figure 1). MiR-148a and miR-126, directly bind to 3ʹ-UTR region of DNA methyltransferases (DNMT1) and inhibit its expression. The third one: MiR-21, indirectly downregulates DNMT1 by targeting RASGRP1 in Ras-MAPK pathway. Expression levels of these miRNAs are significantly increased in CD4^+^ T cells of SLE patients, which as a consequence leads to downregulation of DNMT1 protein levels and to hypomethylation in CD4^+^ T cells [37,38]. These studies suggest that hypomethylation status in CD4^+^ T cells is partly caused by abnormal expression of miRNA and contributes to T and B cells hyperactivity.

It is also clear that miRNAs can regulate the immune response through pathways independent from the DNA methylation machinery. Increased level of miR-126 leads to upregulation of genes encoding autoimmune related proteins such as CD11a and CD70, and by this it contributes to T and B cells autoreactivity [38]. Moreover, miR-21, besides its ability to regulate a methylation in T cells, also affects TCR signaling [39,40,41]. Wang *et al.* [40] observed that miR-21 may augment T cell activation by being involved in induction of activator protein-1 (AP-1) and upregulation of IL-2 expression. Overexpression of miR-21 in normal T cells, isolated from healthy donors, results in acquisition of an activated phenotype [41]. Pre-miR-21 transfected T cells are characterized by enhanced proliferation, increased CD40L expression and interleukin 10 (IL-10) production, and also by a stronger ability to promote autologous CD19 B cell differentiation into plasma cells. On the contrary, the mock-transfected or irrelevant miR-transfected normal T cells do not acquire these features [41]. Silencing miR-21, however, causes the opposite result [41]. Sheedy *et al.* [42] showed that miR-21 is capable of T cells activation control by regulating expression of programmed cell death protein 4 (PDCD4). PDCD4 is a proinflammatory protein that promotes the transcription factor NF-кβ activation and suppresses IL-10. Negative regulation of PDCD4 by miR-21 may contribute, at least partially, to intensification of T cell immune response [42].

Recently, miR-142s were recognized as highly significant in SLE pathogenesis. MiR-142-3p and miR142-5p are both negative regulators of CD4^+^ T cells activation. These miRNAs are responsible for direct inhibition of signaling lymphocytic activation molecule (SLAM)-associated proteins (SAP), CD84 and IL-10. Expression levels of miR-142s in T cells of SLE patients are significantly downregulated. This contributes to increased expression of the aforementioned molecules and to T cells overactivation. The overexpression of SAP and CD84 induces Th2-cytokines production, stimulates follicular Th cells, mediates T/B cells interactions and promotes germinal center formation and antibody production by B cells [43].

Another miRNA involved in T and B cells differentiation and function is miR-181a.; its increased expression enhances mature T cells sensitivity to antigens. On the other hand, the inhibition of its expression in immature T cells leads to reduced sensitivity and impairments during negative and positive selection [44]. Moreover, upregulated expression of miR-181a enhances progenitor B cells differentiation in bone marrow [45]. Although its significance in TCR signaling is evident, it is not clear how exactly miR-181a is involved in pathogenesis of SLE. Although studies show that it correlates with Systemic Lupus Erythematosus Disease Activity Index (SLEDAI) score, there are conflicting data regarding the miR-181a expression level in SLE patients [6,46].

MiR-155 is one of the key regulators of the immune response. Thanks to its ability to target a broad range of genes, it can regulate immune response in various ways. MiR-155 targets include: mRNA of IFNɣRα, Src homology 2 domain which contains inositol 5-phosphatase 1 (SHIP-1), c-Mef, PU.1, SOCS1, and TAB2. It is involved in T cells differentiation, regulation of IFNɣ production, polarization of T cell response towards Th2, maintaining Treg homeostasis, B cells differentiation, activation, survival and regulation of antibody production. It also affects functions and survival ability of DCs [47,48]. Abnormal expression level of miR-155 is observed in many autoimmune disorders. One of them is lupus, where elevated level of miR-155 is observed in B cells. Overexpression of miR-155 may contribute to lupus development by targeting SHIP1, which is an inhibitory signal transducer and its activity leads to inhibition of B cell activation. Downregulation of SHIP1 contributes to enhance B cell survival and higher titer of IgG autoantibodies [49].

MiRNAs also can contribute to SLE pathogenesis through regulation of expression levels of significant cytokines. As mentioned above, T cells in patients with SLE are characterized by IL-2 defects. This deficiency is the reason for decrease in activation of induced cell death and causes prolonged survival of autoreactive immune cells. Abnormal expression level of miR-31 is found to be responsible for defects in IL-2 synthesis in SLE patients. MiR-31 regulates IL-2 production through binding to its target gene—*Rho A*. As a consequence, it enhances promotor IL-2 activation and IL-2 production. Under-expression of miR-31 is positively correlated with decreased IL-2 protein level [50].

Furthermore, it was recently observed that miRNAs influence expression levels of RANTES (regulated on activation normal T cell expressed and secreted). RANTES is responsible for recruiting leukocytes into inflammatory sites. This chemokine plays an important role in pathogenesis of SLE and other autoimmune conditions. MiR-125a directly binds to RANTESs transcription factor—KLF13 and in consequence it down-regulates RATNES expression. MiR-125a level is significantly decreased in T cells in SLE patients. This deficiency directly contributes to the pathological expression of RANTES [51].

MiR-146a negatively regulates the type I IFN pathway. Its under-expression contributes to SLE pathogenesis. Bioinformatics analysis and luciferase reporter assay identified two miR-146a targets—IRF5 and STAT-1, which are key components in the signaling cascade of the type I IFN pathway [30]. Additionally, miR-146a was found to negatively regulate the immune response by targeting tumor necrosis factor receptor associated factor 6 (TRAF6) and IL-1 receptor-associated kinase 1 (IRAK1). TRAF6 and IRAK1 are signal transducers in the NFкβ pathway. MiR-146a contributes to their down-regulation and to the termination of the inflammatory response [52].

Recent studies have suggested that miRNAs play a role in osteoblast differentiation and bone formation. MiR-286 promotes osteoblast differentiation by repression of HDAC (histone deacetylase) expression. Disturbances in miR-286 expression or the presence of polymorphisms within that miRNA might lead to osteoporosis [53].

There is no doubt microRNAs have a huge significance for a proper functioning of the immune system. Any variations of their expression may result in a development of autoimmune diseases. MiRNA expression profiling studies in SLE patients provide novel insights into pathogenic mechanisms and contribute to the development of novel biomarkers for SLE.

## 4. Cytokines as Biomarkers for SLE

Although some tests for diagnosis and monitoring of SLE are already available, there still is a demand to find better markers in order to improve patient care. Better understanding of SLE pathogenesis during the past few years supports an impressive spectrum of potential new SLE biomarkers.

### 4.1. IFN and IFN-Inducible Genes

The type I IFN, prominent candidate biomarker for SLE, is one of the most important endogenous mediators of inflammation and immunity. Dysregulation of IFN in patients with SLE is evident in gene expression profiles, including genes that regulate the type I IFN pathway and IFN-inducible genes. Many of the studies demonstrated below show that aberrations in IFN gene expression signatures are strongly associated with disease activity, as well as with generation of autoantibodies and severe organ complications.

Although, the significance of IFN-α in SLE pathogenesis is well documented, its concentration in serum is very low and hard to detect. Evaluation of IFNs signature genes expression is easier, and more often studied as a SLE biomarker. Nonetheless, there are studies where increased level of IFN-α in serum was reported in patients suffering from severe SLE [54,55]. Increased level of IFN is associated with more severe clinical manifestation and activation of multiple inflammatory cytokines [54]. Serum IFN-α increases markedly at flares and its level correlates positively with the SLEDAI score, level of anti-dsDNA antibodies and IL-10, and inversely with complement components Clq, C3 and leukocytes [55].

In the cross sectional study based on calculated IFN score (IFN score was calculated by measuring expression of INF inducible genes in SLE patients and healthy individuals) it was demonstrated that this score is elevated in about half of studied SLE patients. Higher IFN score correlates with more severe manifestations of the disease involving kidneys, hematopoietic cells, and the central nervous system. Those results suggest that higher IFN gene expression signature may predict a more severe SLE course [56]. Most prominent genes among all IFN-inducible genes for SLE are IP-10 and sialic acid-binding Ig like lectin-1 (SIGLEC-1). IP was detected in 50% and SIGLEC-1 in 86% of studied SLE patients. Both were positively associated with activity of the disease and anti-dsDNA antibody titer, and inversely associated with complement level in serum [57,58]. In order to assess the relation between IFN-regulated gene pattern and clinical outcomes of SLE (such as flares) a long-term study was performed. This study determined the expression level of selected IFNα inducible genes, serological biomarkers and SLEDAI score in periods from 3 to 12 months. The authors reported that, although IFN score is elevated in SLE patients, it does not change significantly during the course of the disease, that is, before, during or after flares. Thus, the value of IFN-signature as biomarker to predict disease flare is greatly limited [59,60].

In another study, Feng *et al.* [61] listed the type I IFN-inducible genes that could serve as good diagnostic biomarkers for SLE. Based on the modified IFN score (integrated expression level of three IFN-inducible genes) and LY6E expression level, it was possible to distinguish SLE from other connective tissue diseases (CTDs) and from healthy controls, with 70%–80% specificity and 70%–80% sensitivity.

Furthermore, the research on IFN induced chemokines revealed them to be promising as SLE disease activity biomarkers. The expression level of chemokines like CXCL11—Interferon-inducible T cell α-chemoattractant (I-TAC), CXCL13—B-lymphocyte chemoattractant (BLC), CXCL10 (IP-10) and CCL3 (MIPα), correlate with disease activity (especially lupus nephritis (LN)) [62]. Also, a calculation of chemokine score, based on a calculation of mRNA expression of seven genes for IFN inducible chemokines: RANTES, MCP-1, CCLl9 (MIP-3B), monokine induced by IFNɣ (MIG), IP-10, I-TAC and IL-8 in peripheral blood cells of SLE patients, does show a correlation with activity of the disease, cumulative organ damage score, anti-Sm antibody and anti-RNP antibody titers. These findings indicate that the chemokine score may serve as a new biomarker for more severe and active SLE disease [63]. Interestingly, longitudinal study showed that chemokine serum levels (IP-10, MCP-1, and MIP-3B) in SLE patients change with flares and remission periods, commensurately with SLEDAI score. Moreover, significantly elevated chemokine serum levels predict flare within one year [64]. In conclusion, monitoring IFN induced chemokines levels in SLE may improve the assessment of a current disease activity, the prediction of a future flare, and therapeutic decision-making.

IFN, due to its significant meaning in pathogenesis of SLE, represent a potentially important target for new treatment therapies. Consequently, several new therapies targeting IFN have been developed and tested. IFN-ĸ-kenoid is a potential new drug for SLE treatment. Its application induces polyclonal antibodies that neutralize all subtypes of human alpha interferons (huIFNα) and inhibits disease progression in mice [65,66]. Also, there are studies of monoclonal antibodies that bind to and neutralize IFN like Sifalimumab (MEDI 545) and Rontalizumab (RG7415). Those studies confirm significant influence of IFNα blockade on expression of IFN inducible genes in SLE patients, but so far they do not show consistent effect in a clinical manifestation of the disease [67,68].

### 4.2. IL-6

Another important cytokine studied as a potential biomarker for SLE disease is IL-6. This cytokine is a key regulator of hematopoiesis and immune defense. Disturbances of its expression are involved in the pathogenesis of SLE and other autoimmune diseases. In SLE, its serum and urinary levels are significantly elevated and correlate with disease activity, anti-dsDNA, ESR and CRP [69,70,71]. High level of IL-6 is also observed in bronchoalveoar lavage fluid (BALF) of SLE affected patients, and correlates with disease activity [72]. Although these findings highlight the pathological role of IL-6 in SLE, the results of the existing studies are sometimes inconsistent [73]. This may be due in the fact that the profile of cytokines can change depending on phenotype and range of the disease or in the nature of these studies [74]. Cytokine levels in SLE patients differ from healthy individuals. Also, its balance changes in distinct SLE phenotypes. For example, IL-6 concentration in cerebrospinal fluid of SLE patients with nervous system affectations is higher than in patients without these symptoms [75]. It indicates that this cytokine balance may be important to describe phenotype of disease or severity of symptoms but not to predict susceptibility to SLE.

Blocking IL-6 and IL-6R, using specific antibodies, prevents the onset and progression of SLE in the mice model [76]. IL-6 knock-out mice show milder symptoms and prolonged life in murine SLE [77]. *In vivo* studies also confirm that IL-6R blockade leads to decreased activity of B and T cells [78].

Some of the available drugs, routinely used in SLE treatment, target IL-6. For example, corticosteroids inhibit IL-6 production at the transcription level [79], whereas NSAIDs inhibit translation of IL-6 and its activity [80]. Also, several new potential therapeutics, targeting IL-6, have been developed. Tocilizumab administration results in decrease in number of tender and swollen joints, reduction of joint damage [81], and in diminishing the number of harmful neutrophils, that accumulate in synovium [82]. Tocilizumab reduces CRP serum concentration [83], and decreases the number of Th17 cells [84]. A different potential drug for inflammatory diseases that targets IL-6 activity is Sirukumab, which inhibits IL-6 phosphorylation by STAT 3, neutralizing its activity [85].

### 4.3. B Lymphocyte Stimulator Protein (BLys)

BLys, also known as BAFF, is a key growth factor for B cells. Its mRNA level and protein concentration in serum are higher in SLE patients than in healthy controls and its excess is observed in about 30% of patients [86,87]. Although serum BLyS level generally correlates with anti-dsDNA concentration, the change in serum BLyS level does not correlate with change in disease activity in the individual patient [87]. However, Petri *et al.* [86] reported, that BLys level in plasma, measured at the previous visit, correlates with the higher disease activity score (Safety of Estrogens in Lupus Erythematosus—National Assessment—SELENA) on subsequent visit. Those results suggested that BLys concentration may be related with the future development of the disease [86]. Although elevated serum BLyS phenotype is not associated with a specific organ involvement, [87] some studies indicate that renal lupus patients have higher levels of serum BLyS compared to SLE patients without renal manifestations [88,89]. Low BLyS level measured at a baseline, in LN patients, predicts good response to therapy. Parodies *et al.* [90] indicated that in LN patients, BLyS level <1.5 ng in serum predicts both clinical and histopathological improvement after therapy, with a 92% positive predictive value (PPV). BlyS may be a potential biomarker of renal disease activity in lupus and a candidate predictor of treatment response in LN.

Interestingly, a new SLE therapy that targets the BLys signaling pathway has been approved recently. Belimumab is a human immunoglobulin G1λ, capable to inhibit B cell survival by neutralization of BLys. It is used as an add-on to standard therapy. Belimumab significantly improves SLE response index (SRI), inhibits production of autoantibodies, reduces disease activity and does not affect the deterioration of patients’ health [91,92,93].

### 4.4. A Proliferation Inducing ligand (APRIL)

Similarly to BAFF, a member of the TNF superfamily—APRIL—is important for B-cell survival and maintenance. APRIL was found to be involved in the pathogenesis of Neuropsychiatric SLE (NPSLE) [94]. Its protein level is increased in cerebrospinal fluid (CSF) of NPSLE patients compared to other neurological diseases sufferers. Moreover, it was observed that APRIL level in CSF correlates positively with fatigue, highlighting the value of this molecule in NPSLE pathogenesis [94]. APRIL is also a good biomarker of renal disease activity in LN patients. Its level is significantly elevated in LN serum compared to controls. Furthermore, the level of APRIL decreases in response to therapy. Interestingly, this decline is observed only in serum of patients who respond to treatment [90]. Treamtrakanpon *et al.* [95] showed that APRIL may be used as a biomarker of treatment response in LN. High serum APRIL and internal mRNA levels are associated with resistance to treatment. Serum level of APRIL lower than 4ng/ml indicates good response to treatment with a sensitivity of 65% and specificity of 87.5% (PPV of 93% and negative predictive value (NPV) of 54%) [95].

### 4.5. IL-10

IL-10 being an important immunoregulator that inhibits T cells function and suppresses proinflammatory cytokines, forms another potential biomarker for SLE disease [96,97]. Its increased production by monocytes and lymphocytes is related with clinical and serological indices of disease activity and with elevated ANAs production, especially anti-dsDNA antibody titer. These data suggest that IL-10 has an ability to promote humoral immune responses. It enhances MHC class II expression on B cells and induces Ig production. In SLE patients, IL-10 level in serum is elevated and correlates positively with ESR, anti-dsDNA antibody titers but negatively with complement fraction C3d [98]. Gröndal *et al.* [73] confirmed the association between IL-10 level in serum and anti-dsDNA antibody titer but did not observe any correlation between increased level of IL-10 and disease activity. On the other hand, Sun *et al.* [99] suggested that IL-10 has a significant role in SLE pathogenesis, by being involved in DCs differentiation and function. IL-10 levels in BALF and exhaled breath condensate (EBC) are significantly increased in SLE patients compered to control groups. This growth is probably caused by enhanced production of IL-10 by monocytes and B cells [72].

### 4.6. IL-17 and IL-23

IL-17 is a pleiotropic cytokine, which plays a role not only in tissue inflammation, but also enhances viral replication. It is known that IL-17 has a crucial role in survival and proliferation of B cells. IL-23 is important for Th-17 cells maintenance, and in consequence it has strong influence on expansion of IL-17 [100]. Over-production of these proinflammatory cytokines is found in SLE serum and kidney biopsies [101,102]. Overall, the inflammatory status of SLE patients is characterized by high production of IL-17. Higher IL-17 concentration is connected with TCRαβ^+^CD4-CD8-T cells, which have been shown to infiltrate the kidney in LN. Those cells are highly expanded and able to produce significant amounts of IL-17 [103]. Also, high level of IL-17 in serum at baseline may serve as a biomarker of an inadequate histopathological outcome, after immunosuppressive therapy in LN [104].

### 4.7. Tumor Necrosis Factor (TNF)

TNF is a proinflammatory cytokine and a B cell growth factor. Its function in the pathogenesis of SLE may vary depending on genetic background, organ involvement and severity of the disease. TNF blockade transiently increases the level of autoantibodies that bind chromatin and phospholipids. At the same time, open-label data suggest that TNF blockade suppresses inflammatory manifestations of SLE, and long-term benefits are seen in patients with LN [74]. There are studies that confirm elevated level of this factor in SLE patients [105], increased level of mRNA, and correlation between TNF and disease activity in a murine model of SLE [106,107]. On the other hand, TNF deficient mice develop more severe symptoms during the course of SLE [108]; these inconsistent results might indicate that TNF function in SLE pathogenesis differs depending on the phenotype of manifestations, and severity of the disease.

### 4.8. IL-12

IL-12, as a proinflammatory and immunomodulatory cytokine, is a master regulator of the polarization of immune responses to Th1. Thus it forms a connecting point between cellular and humoral branches of innate resistance and antigen-specific adaptive immunity. It promotes IFN-γ production as well as the cytolytic activity of T and natural killer (NK) cells. IL-12 induces cellular immunity and stimulates an antiangiogenic program mediated by IFN-γ-inducible genes and by lymphocyte-endothelial cell cross-talk [109]. Although, Horwitz *et al.* [96] suggest that its level is often low in patients with recent onset of SLE, other studies shows that it is elevated in both serum and urine of patients with LN [101,110], suggesting that higher concentration of IL-12 may partially contribute to pathogenic inflammation and LN development.

### 4.9. Limitation of Cytokine Measurement

There are a number of features and conditions that can influence cytokine production. Multiple studies discuss differences in cytokine production associated with donor age. Longitudinal cytokine measurements in pediatric and adult patients identified multiple differences between these two groups in terms of proinflammatory cytokines (IL-6, IL-8, IL-1α IL-1β MCP-1 MIP-1α IL-15, IL-5 IL-17 IL-18 and IP-10) and anti-inflammatory cytokines such as IL-10 G-CSF, IL-13 IFN-γ and IL-4 [111]. Furthermore, immune defense capacity differs between men and women. Women develop autoimmune diseases more commonly. This suggests that sex also affects the level of produced cytokines. Another factor that influences cytokine level measurement is that cytokines present a circadian pattern. Some of them exhibit diurnal rhythms with a peak in the morning and are related to the rhythm of plasma cortisol and melatonin. Circulating cytokine levels can be affected by many more factors like diet, physical exercise or stress. Sample handling may also influence the measurement of cytokines.

Those factors do not concern all cytokines levels but should be considered if validating a biomarker for clinical use [111].

## 5. MicroRNA as Biomarkers for SLE

MiRNAs are essential regulators of the immune responses and autoimmunity. Recent studies have revealed an extremely important role of miRNAs in SLE, thereby providing new insights into lupus pathogenesis. The levels of miRNAs in serum are stable, reproducible, and consistent among individuals of the same species. There is evidence that serum miRNAs contain fingerprints for various diseases, for example, circulating miRNA patterns could be used to distinguish SLE from other inflammatory diseases. Alterations of miRNAs expression in body fluids may serve as potential biomarkers for SLE (Table 1). Monitoring serum biomarker levels in SLE may improve the assessment of disease activity, help to predict future flares, and improve the overall clinical decision-making in process of SLE treatment.

### 5.1. MiR-146a

MiR-146a is responsible for down regulation of the innate immune response, and is underexpressed in SLE CD4^+^ T cells. Impaired expression of mi-146a leads to alterations in the type I IFN pathway and is inversely correlated with disease activity [112]. Moreover, single nucleotide polymorphisms (SNPs) in promotor regions of miR-146a, are associated with the onset and progression of SLE [113]. Mi-146a expression levels in serum negatively correlate with disease activity and degree of proteinuria [112,114]. It was also observed that treatment with steroid doesn’t influence already altered expression of miR-146a, suggesting that miR-146a is intrinsically underexpressed in lupus patients [113]. Wang and colleagues [114] reported that miR146a level in serum of SLE patients is significantly reduced but miR-146 level in urine is elevated. A different study also confirms that expression of miR146 level in glomerulus is elevated in LN patients, and is associated with glomerular filtration rate (GFR) and histological activity index [114]. The strong association between the level of miR-146a and clinical disease activity indicate that it may serve as new disease biomarker.

### 5.2. MiR-125a

MiR-125a expression level is different in SLE patients compared to controls. Its expression is significantly downregulated in CD4^+^ T cells. Reduced level of miR-125 leads to the RANTES overproduction, contributing to the disease pathogenesis [51]. Wang *et al.* [115] showed that miR-125a level in blood is only downregulated in patients with SLE compared to healthy controls and Rheumatoid Arthritis (RA) patients. Moreover, changed expression of miR-125a in urine supernatant of LN children is strongly associated with SLEDAI score. It is higher in active LN than non-active LN and also moderately correlates with traditional LN biomarkers like GFR and creatinine ratio. The exploratory regression modeling describes miR-125 level in urinary supernatant as a relevant and accurate biomarker of LN activity [116].

### 5.3. MiR-126, MiR-21 and MiR-148a

The DNA-methylation-associated miRNA—miR-126—is significantly deregulated in peripheral blood mononuclear cells (PBMCs) of SLE patients. Its expression level is much higher in the blood of patients compared to healthy controls and it correlates negatively with DNMT1 level [37]. Interestingly, opposite changes are noted in miR-126 level expression in blood of RA patients. Those results suggest that miR-126 may serve as lupus specific biomarker [115].

Alike miR-126, miR-21 is highly upregulated in blood of SLE patients [38,115]. Level of miR-21 is elevated especially in T cells and its expression is associated with SLEDAI score of lupus patients [38]. Stagakis *et al.* [41] proved that miR-21 is differently expressed between patients with an active and inactive SLE. In this study, not only its expression correlated with SLEDAI score but also longitudinal analysis of two patients showed significant decrease in miR-21 during the remission of the disease [41]. Interestingly, miR-21 level is also upregulated in RA patients compared to healthy controls. These results suggest that it is strongly involved in the pathogenesis of autoimmune diseases but is not specific for SLE [115].

The level of miR-148a is remarkably increased in human lupus CD4^+^ T cells compared to healthy controls. It is also positively correlated with SLEDAI score. Expression level of this microRNA does not differ between patients with LN and without. Moreover, treatment with steroids does not influence expression level of miR148a in lupus T cells [38].

Those DNA-methylation associated miRNAs contribute to SLE pathogenesis through affecting proper lymphocytes functions. Their expression levels are strongly correlated with clinical activity of lupus. This suggests the potential use of miR-126, miR-21 and miR-148a as disease biomarkers and novel therapeutic targets for SLE.

### 5.4. MiR-142-3p and MiR-142-5p

Recent studies showed that negative regulators of CD4^+^ T cells activity—miR-142-3p and miR-142-5p—are significantly downregulated in lymphocytes of lupus patients and that no treatment (corticosteroids, antimalarial or immunosuppressive agents) affects expression level of either miR-142-3p or -5p. Although expression miR-142 level is changed in SLE patients, it does not correlate with SLEDAI score [43]. In a different study, Carlsen *et al.* [6] observed that miR-142-3p circulating in plasma is significantly increased in SLE patients compared to healthy subjects. They suggest increased exocytosis effects in decreased intracellular content of this miRNA [6]. The search for LN biomarkers provided similar data about expression levels of these microRNAs. MiR142-5p in renal biopsies was found to be up-regulated in LN patients compared to the normal control group [117]. Although miR-142 is significantly implicated in SLE pathogenesis, present data do not indicate that this miRNAs may be useful as a diagnostic biomarker of SLE or LN.

**Table 1:** Biomarkers involved in systemic lupus erythematosus.

Biomarker	Expression in Lupus	Correlation with Lupus Activity and SLEDAI	SLE Disease Association	Ref.
IFN	↑ (serum)	Positively correlated	Increase expression of auto-antigens, Central nervous system (*CNS*) lupus, fever	[54,55]
IFN inducible genes	↑ (serum, urine)	Positively correlated with SLEDAI, correlated with flares and remission periods of SLE	More severe SLE course (CNS, hematologic and renal manifestations)	[56,57,58,59,60]
IL-6	↑ (serum, urine, BALF)	Positively correlated with SLEDAI	Lupus nephritis, increase anti-dsDNA level, CNS	[69,70,71,72]
BLyS	↑ (serum, plasma)	Positively correlated with SELENA	Increase anti-dsDNA level, not associate with specific organ system involvement	[86,87,88,89]
IL-10	↑ BALF	Not significantly correlated with disease activity	Increase anti-dsDNA	[72,73,98]
IL-17	↑ (serum, kidneys)	Correlated with disease activity	Lupus nephritis	[101,102]
TNF	↑ (serum, kidneys)	Correlated with disease activity?	Lupus nephritis	[106,107]
IL-12	↑ (serum, urine)		Lupus nephritis	[110,111]
miR-146a	↓ (CD4^+^ T cells, serum) ↑ (urine)	Inversely correlated with disease activity	Proteinuria, lupus nephritis, GFR, histological activity index	[112,113,114]
miR-125a	↓ (CD4^+^ T cells, urine)	Inversely correlated with SLEDAI score	Lupus nephritis (GFR and creatinine ratio)	[51,115,116]
miR-126	↑ (PBMCs)	Positively correlated with disease activity	Induces DNA hypomethylation , not associate with specific organ system involvement	[37]
miR-21	↑ (PBMCs)	Positively correlated with SLEDAI score, correlated with flares and remission periods of SLE	Induces DNA hypomethylation , not associate with specific organ system involvement	[38,41,115],
miR-148a	↑ (PBMCs)	Positively correlated with SLEDAI score	Induces DNA hypomethylation , not associate with specific organ system involvement	[38]
miR-142	↓ (PBMCs) ↑ (plasma, kidneys)	Not correlated with SLEDAI score	Inhibit T cell activity, not associate with specific organ system involvement	[6,43,117]
miR-181a	↑ (plasma), ↓ (blood)	Positively correlated with SLEDAI score	Not associate with specific organ system involvement	[6,46]

↑—increased level of biomarker, ↓—decreased level of biomarker.

### 5.5. MiR-155

Abnormal expression patterns of miR-155 characterize many pathologic conditions like cancers, hematological malignancies, viral infections and autoimmune diseases. It is probably due to the fact that it has functional relevance in the biology of lymphocytes [47]. There is contrary evidence concerning miR-155 expression level in SLE patients. Some studies showed that its level is significantly elevated in B and T cells [49,118]. On the other hand, two other studies, reported that concentration of miR155 in SLE patients is significantly reduced [115,119]. These findings indicate that miR-155 affects a broad range of mechanisms and can contribute to pathogenesis of the disease in different ways.

## 6. Conclusions

The pathogenesis of SLE is very complicated. Disease development depends on many factors and on disruption of mechanisms and signaling pathways, essential for the proper functioning of the immune response. Growing evidence indicate the pathogenic role of cytokines and miRNAs in SLE pathogenesis. MiRNAs and cytokines may be available as new important biomarkers for SLE. Many of them were found to have significant meaning in SLE development. However, there are still many inconsistent data which need to be clarified before these factors can be used. Better understanding of the cytokines and miRNAs importance in the immune response will allow for the creation of new biomarkers and therapeutic agents useful in an earlier diagnosis and improved treatment of SLE.
